# Early diagnosis and tailored treatment in atypical idiopathic thrombocytopenic purpura: A CARE compliant case report

**DOI:** 10.1097/MD.0000000000044263

**Published:** 2025-09-05

**Authors:** Tirath Patel, Monica Kharat, Jabez David John, Hitanshi Panchal, Nana Sardarova, Gayatri Misra, Muhammad Ahmed, Richard M. Millis

**Affiliations:** a Trinity Medical Sciences University School of Medicine, Kingstown, Saint Vincent and the Grenadines; b Department of Medicine, Icahn School of Medicine at Mount Sinai, Queens Hospital Center, New York, NY; c Malla Reddy Institute of Medical Sciences, Hyderabad, India; d GMERS Medical College and Hospital, Gotri, Vadodara, India; e Henry Ford Warren Hospital, Warren, MI; f American University of Antigua, Osbourn, Antigua and Barbuda; g Henry Ford Macomb Hospital, Clinton Township, MI; h Department of Pathophysiology, American University of Antigua, Osbourn, Antigua and Barbuda.

**Keywords:** case report, idiopathic thrombocytopenic purpura, ITP

## Abstract

**Rationale:**

Idiopathic thrombocytopenic purpura (ITP) is a hematological disorder characterized by a decrease in platelet count due to increased destruction or decreased production. Although the pathophysiology and etiology remain largely unknown, understanding the typical and atypical presentations of ITP is crucial for early diagnosis and effective management. This case report highlights the rationale behind a comprehensive approach for the diagnosis and treatment of ITP, especially in cases with atypical presentations.

**Patient concerns:**

A 45-year-old woman presented with a mucocutaneous petechial rash spreading over the ocular and oral areas of the face, accompanied by similar manifestations in the limbs. While petechiae are a hallmark of ITP, the initial widespread distribution and specific involvement of these extensive mucocutaneous areas were considered atypical presentation patterns in this case. She also reported moderate gum bleeding, epistaxis, and spontaneous ecchymosis of the oral mucosa. These symptoms suggested a potential platelet disorder.

**Diagnoses:**

Based on the clinical presentation and laboratory findings, the diagnosis of ITP was made. The patient’s symptoms and laboratory results were consistent with the typical features of ITP, including a decreased platelet count, petechiae, and manifestations of bleeding.

**Interventions:**

A thorough history and physical examination were conducted to rule out other potential causes of thrombocytopenia, including infections, medications, and underlying autoimmune diseases. Laboratory tests, including complete blood count, peripheral blood smear, and coagulation profile, were performed to assess platelet count, morphology, and clotting function. The diagnosis was initially followed by conservative management. Later on, the patient was also treated with corticosteroids and then intravenous immunoglobulin.

**Outcomes:**

The patient responded well to intravenous immunoglobulin, thereby demonstrating the effectiveness of the treatment. She was then discharged with maintenance doses of corticosteroids and a close follow-up schedule.

**Lessons:**

This case report illustrates the importance of recognizing the diverse presentations of ITP, including its atypical manifestations. Early diagnosis and effective management are crucial for improving patient outcomes. A comprehensive approach, including thorough history, physical examination, and laboratory tests, is essential for the accurate diagnosis and effective treatment of ITP.

## 1. Introduction

Idiopathic thrombocytopenic purpura (ITP) is an autoimmune syndrome characterized by abnormally low level of platelets, with the majority of European studies showing that ITP affects approximately 5 in every 100,000 children and 2 in every 100,000 adults annually.^[1–3]^

In the United States, the annual incidence of ITP is estimated to be approximately 3.3 cases per 100,000 people. Across Europe, the incidence of adult cases varies, with estimates ranging from 1 to 4 cases per 100,000 individuals annually. In Northern Europe, the incidence is approximately 2.68 per 100,000 people.^[4]^ Denmark has an annual incidence of 2.25 per 100,000 individuals.^[4,5]^ In France, the estimated annual incidence is 2.9 per 100,000 people, influenced by factors such as age, season, and geographic location.^[1,6]^ In the United Kingdom, the annual incidence is approximately 1.6 per 100,000 individuals.^[4,7]^ ITP is characterized by the premature destruction of platelets. This occurs when autoantibodies bind to platelet antigens, leading to the removal of the platelets by the reticuloendothelial system, primarily in the spleen, and the immune cells mistakenly identify the platelets as pathogens, producing autoantibodies. This destruction of platelets leads to a significantly higher risk of bleeding, as platelets play a vital role in hemostasis. The exact pathophysiology remains idiopathic, but the etiology is strongly associated with infections such as HIV, *Helicobacter pylori*, and Hep-C; there is also a strong association with drug-onset ITP.^[8]^ Petechiae, purpura, easy bruising, and bleeding are the typical signs of ITP.^[8–11]^ These manifestations can persist for various periods. Based on the duration of symptoms, ITP is classified as acute (0–3 months), persistent (3–12 months), and chronic (>12 months).

Most patients with ITP do not exhibit pathognomonic (specifically indicative) or obvious symptoms that clearly confirm the diagnosis. Typically, comprehensive testing is a prerequisite for the proper diagnosis of ITP, as it is a diagnosis of exclusion. Most commonly, a simple complete blood count and peripheral blood smear, along with clinical suspicion, lead to testing for a detailed history of drug-induced ITP, extensive testing to exclude infections that induce ITP, and secondary causes that may induce ITP. Delay in diagnosis or missing the diagnosis can have devastating effects, leading to severe hemorrhagic complications, such as intracranial hemorrhage, gastrointestinal hemorrhage, or heavy menstrual bleeding. The standard management plan for ITP involves the use of either one or a combination of corticosteroids and immunotherapies. In severe cases, therapeutic splenectomy can be considered.^[12,13]^

While the classical presentation of ITP is well-documented, there is a significant knowledge gap in the recognition and tailored management of ITP manifesting with atypical features, which causes a diagnostic challenge, potentially leading to delays in treatment and an increased risk of complications. This case report aims to address this gap by detailing an instance of ITP with an atypical mucocutaneous presentation, emphasizing the diagnostic approach, and administering a tailored treatment.

We report a case of ITP, highlighting the challenges in its detection and management, as well as customized treatment plans. Understanding the various ITP presentations will enhance patient management by facilitating early diagnosis and improving outcomes in this complex autoimmune condition.

## 2. Methods

As the patient information was de-identified and the results of this study are not generalizable, ethical clearance was not required for this case report.^[14]^ The patient provided informed consent for the case report and publication.

### 2.1. Case report

#### 2.1.1. Case introduction

A 45-year-old woman presented to the clinic with a mucocutaneous petechial rash covering the eye, mouth, and peripheral limbs that had persisted for the past month. The patient was asymptomatic 1 month ago and insidiously developed several petechiae and purpura over the conjunctivae, oral mucosa, and peripheral limbs. This was associated with moderate gum bleeding, epistaxis, and spontaneous ecchymosis in the oral mucosa. The patient denied any blood in stools, hematuria, burning micturition, recent infections, drug consumption, or a history of hematologic abnormalities.

**Figure 1.**
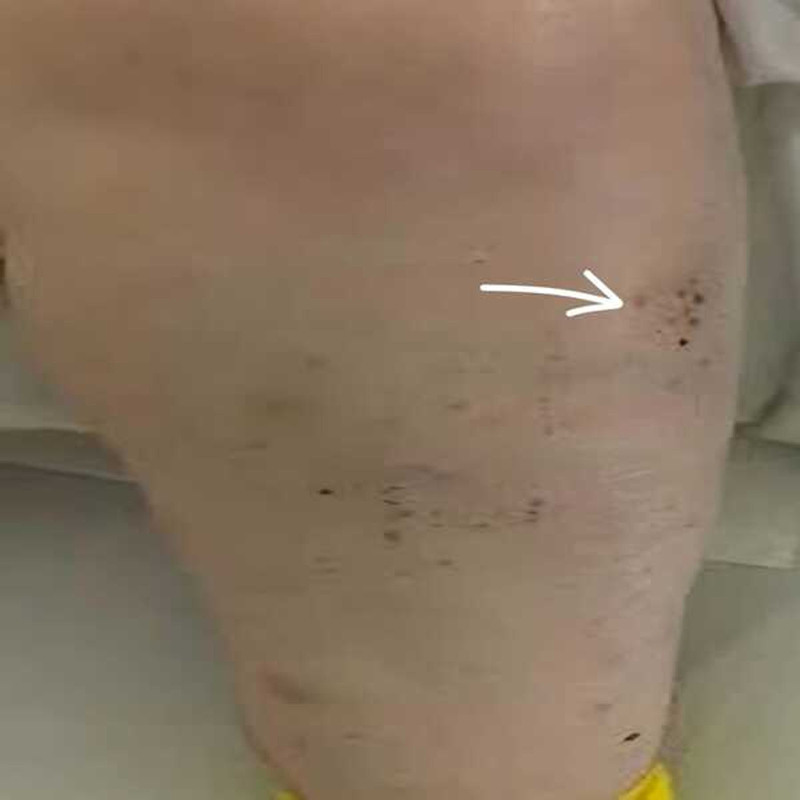
The arrow is showcasing petechiae in the extremities. Rash consists of tiny pinpoint-sized spots (1–2 mm in diameter) that are flat to touch and non-blanching (do not fade when pressure is applied). The color ranges from bright red to purple or brown depending on the duration and extent of bleeding into the skin.

Physical examination revealed a mucocutaneous petechial rash with hemorrhagic bullae covering the conjunctivae, oral mucosa, and extremities (Fig. 1). In addition, spontaneous ecchymosis was observed inside the cheeks. No evidence of organomegaly was noted on either physical examination or ultrasonography, and there was no evidence of lymphadenopathy.

Petechial rash is a hallmark clinical manifestation of immune thrombocytopenia purpura caused by bleeding into the skin due to a severely reduced platelet count. The rash provides a key diagnostic clue and is typically observed in patients when platelet counts fall below 30 × 10⁹/L, as shown in this patient, represented by Figure 1. Petechiae are commonly found in areas subject to dependent positioning or pressure, such as the lower extremities (e.g., ankles, shins, and feet), due to gravity-induced pooling of blood. Pressure points around elastic bands (e.g., waistbands and socks). Rash can also be widespread, including in the trunk, arms, face, and mucous membranes (e.g., inside the mouth), especially in severe cases. Petechiae may appear as isolated dots or clusters, often coalescing into a speckled pattern that overlies the affected areas. Unlike other rashes, petechiae were not associated with itching or raised lesions. Purpura are larger, confluent areas of bleeding (>2 mm), which may accompany petechiae in patients with more severe thrombocytopenia. Petechiae are often observed alongside oral mucosal bleeding (e.g., on the hard palate or buccal mucosa). Differentiating features of ITP rash include non-blanching, wherein petechiae do not fade when pressure is applied (distinguishing them from erythema or inflammatory rashes), and the absence of systemic symptoms, wherein petechial rash in ITP is not associated with fever, pruritus, or systemic signs of infection (unlike vasculitis or infectious causes of petechiae). The pathophysiological mechanisms of ITP rash involve a very low platelet count that is insufficient to maintain vascular integrity, leading to spontaneous leakage of blood into the dermis and increased capillary fragility, where in the absence of adequate platelet plugs, minor trauma or increased venous pressure in dependent areas causes blood to seep through capillary walls.

#### 2.1.2. Investigations

Laboratory workup revealed severe thrombocytopenia, with a platelet count of 10,000/mm³ (the standard range is 150,000–400,000/mm³), but no other abnormalities in the complete blood count. Examination of the peripheral blood smears revealed thrombocytopenia, but no morphological deformities or indications of clumping or dysplasia were noted. Viral marker serologies were negative, and the coagulation profile, liver function, and renal function tests were all within normal ranges. Antinuclear antibody, rheumatoid factor, and anti-double-stranded DNA tests, performed as part of an autoimmune workup, yielded negative results. To exclude vitamin B12 and folate deficiency as a cause of thrombocytopenia, Vitamin B12 and B9 levels were worked up and revealed to be normal. Additionally, intrinsic factors in the castle were normal. A bone marrow biopsy ruled out any thrombocytopenia-related reasons, and ITP was identified based on comprehensive clinical presentation, test results, and exclusion of other causes. The thoroughness of the diagnostic process instills confidence in the accuracy of the diagnosis and the effectiveness of the treatment plan. In addition to isolated thrombocytopenia and absence of underlying causes or other autoimmune diseases, the patient met the diagnostic criteria for ITP.

#### 2.1.3. Treatment and results

The patient was initially managed conservatively, with close observation. However, corticosteroids (oral dexamethasone at 40 mg/day for 4 days, along with 1 g/day methylprednisolone IV for 3 days and close intravenous immunoglobulin [IVIG] therapy) were started at 1 g/kg, as severe thrombocytopenia persisted and recurrent epistaxis developed on the 2nd day, for a period of 3 days. The patient’s outcome showed an increase in platelet count to 100,000/mm^3^ within 5 days of observation, and the patient responded well to IVIG, demonstrating the effectiveness of the treatment. Following discharge, she was administered oral corticosteroids at a tapering dose for maintenance medication and a close follow-up schedule.

Table 1 shows a summary of all laboratory tests performed on the patient.

**Table 1:** Summary of lab work.

Test category	Result	Reference range
General hematology		
White blood cell count	7.5 × 10³/µL	4.0–10.0 × 10³/µL
Hemoglobin	14.2 g/dL	13.0–17.0 g/dL
Mean corpuscular volume	92.5 fL	83.0–101.0 fL
Mean corpuscular hemoglobin	30.8 pg	27.0–32.0 pg
Platelet count	10,000	150–410 × 10³/µL
Reticulocyte count	1.80%	0.5–2.5%
Coagulation		
Prothrombin time (PT)	12 seconds	11–13.5 seconds
Activated partial thromboplastin time (aPTT)	32 seconds	25–35 seconds
Liver function tests (LFTs)		
Bilirubin (total)	0.7 mg/dL	0.3–1.2 mg/dL
Bilirubin (direct)	0.1 mg/dL	0.0–0.3 mg/dL
ALT	20 U/L	Up to 33 U/L
AST	24 U/L	Up to 32 U/L
Albumin	4.2 g/dL	3.4–5.4 g/dL
ALP	90 U/L	44–147 U/L
Kidney function test		
Creatinine	0.8 mg/dL	0.6–1.2 mg/dL
Electrolytes		
Potassium	4.2 mosm/L	3.6–5.2 mosm/L
Chloride	101 mosm/L	96–105 mosm/L
Sodium	141 mosm/L	135–145 mosm/L
Urinalysis		
Appearance	Normal	Clear/light yellow, no odor
Specific gravity	Normal	1.018
pH	6	6 (normal: 6–7)
Protein	Normal	<150 mg/dL
Viral markers serology		
HIV	Negative	Negative
Hepatitis C (Hep-C)	Negative	Negative
Autoimmune workup		
ANA	Negative	Negative
RF	Negative	Negative
Anti-dsDNA	Negative	Negative
Misc.		
Vitamin B12	455 pg/mL	200–900 pg/mL
Folate (vitamin B9)	2.7 ng/ml	>3.9 ng/mL
Intrinsic factor	1.35	1.21–1.52 AU/mL
Bone marrow biopsy	Normal, no cause for thrombocytopenia identified	
Uric acid	4 mg/dL	2.5–7.0 mg/dL
Peripheral blood smear	Thrombocytopenia, no abnormalities noted	

ALP = alkaline phosphatase, ALT = alanine aminotransferase, ANA = anti-nuclear antibody, aPTT = activated partial thromboplastin time, AST = aspartate aminotransferase, HIV = human immunodeficiency virus, LFTs = liver function tests, MCH = mean corpuscular hemoglobin, MCV = mean corpuscular volume, PT = prothrombin time, RF = rheumatoid factor, WBC = white blood cell.

## 3. Discussion

### 3.1. Clinical insights

ITP is an autoimmune disease that causes thrombocytopenia via immune-mediated platelet destruction. An increased propensity to bleed, which presents as petechiae, purpura, ecchymosis, or mucocutaneous bleeding, is the defining feature of ITP.^[8,15]^ However, the case of a 45-year-old female patient with an early diagnosis of ITP highlights better outcomes that create, on the contrary, clinical suspicion that can overcome the diagnostic hurdles and allow early specialized therapy approaches. The relevance of taking into account alternate manifestations of ITP is that increased knowledge can guide clinicians to suspect ITP and allow for early diagnosis of ITP, even though it can manifest as an atypical appearance of broad petechial rash throughout the extremities, oral mucosa, and conjunctivae, as well as scattered ecchymoses, in the absence of typical bleeding symptoms, emphasizing the clinical variety of the illness, requiring an extensive investigation to rule out other possible causes of thrombocytopenia and a broad differential diagnosis. In this instance, thorough laboratory tests, including viral serologies, coagulopathies, and other autoimmune diseases, were performed to exclude underlying infections, coagulation profiles, and autoimmune markers.^[16]^

### 3.2. Pathophysiology

ITP is a multifactorial autoimmune disease. At the molecular level, ITP pathogenesis involves complex interactions between autoantibodies, cytotoxic T cells, dysregulated immune checkpoints, and defects in megakaryocyte function. The hallmark of ITP is the presence of autoantibodies against platelet surface glycoproteins such as GPIIb/IIIa and GPIb/IX.^[17]^ Complement activation via the classical pathway may contribute to platelet destruction in some patients. Cytotoxic T lymphocytes (CTLs) play a critical role in the direct destruction of platelets and megakaryocytes. Platelet antigens present on MHC class I molecules are recognized by CTLs, leading to targeted lysis. CTLs release perforin and granzymes, inducing the apoptosis of platelets and megakaryocytes.^[18]^ The Fas/FasL pathway is also thought to contribute to platelet apoptosis in ITP patients. Defects in immune tolerance mechanisms contribute to autoimmunity in ITP, driven largely by dysfunctional T-cell subsets. Increased Th1 and Th17 cells secrete pro-inflammatory cytokines such as IFN-γ, IL-17, and IL-21, which amplify immune responses. Reduced T regulatory cells impair the suppression of autoimmunity, and overexpression of the transcription factors T-bet (Th1) and RORγt (Th17) is reported to correlate with the severity.^[19]^ Autoantibodies and CTLs also target megakaryocytes, leading to defective platelet production. Antibody-mediated inhibition of thrombopoietin signaling disrupts megakaryocyte maturation, and CTL-induced apoptosis of megakaryocytes reduces platelet production. These events appear to be associated with dysregulated PI3K/AKT/mTOR signaling pathways, impairing megakaryopoiesis in ITP.^[20]^ Elevated levels of pro-apoptotic molecules such as Bax and caspase-3 enhance megakaryocyte apoptosis. Dysregulation of the intrinsic apoptotic pathway has been shown to induce mitochondrial depolarization and caspase activation. Increased expression of the pro-apoptotic proteins Bak and Bax, in combination with decreased expression of the anti-apoptotic protein Bcl-2, has been observed in ITP platelets.^[21]^ Mitochondrial dysfunction and reactive oxygen species are also likely to play a role in the apoptosis of platelets. Pro-inflammatory cytokines appear to exacerbate autoimmune and inflammatory conditions in patients with ITP. Elevated levels of IL-6, TNF-α, and IL-1β contribute to chronic inflammation and antibody production, whereas decreased production of IL-10 may explain the failure to suppress autoimmunity in ITP. Evidence is emerging that failure to regulate immune cell cycle checkpoints such as PD-1/PD-L1 and CTLA-4 pathways, combined with abnormal expression of miRNAs targeting genes involved in T cell and B cell regulation, contributes to the loss of immune tolerance.^[22]^ Moreover, epigenetic influences, including DNA methylation and histone modifications, affect gene expression in immune cells and are likely to account for the environment–gene interactions associated with autoimmunity in ITP.

### 3.3. Diagnostic approach

After the patient’s initial assessment and stabilization, clinical examination revealed mucocutaneous purpuric lesions, and an initial blood workup revealed thrombocytopenia. The peripheral smear confirmed thrombocytopenia, assessed platelet morphology, and ruled out other blood disorders. The coagulation profile workup revealed normal clotting factors, indicating a need for further investigation of the etiology. However, it increased the bleeding time due to a lack of platelets, further clarifying the increased destruction of platelets and further assessing kidney and liver function tests to check for any viral etiologies affecting these organs.

The autoimmune profile revealed no reason to suspect an autoimmune disorder; normal Vitamin B12 and B9 levels, along with normal intrinsic factors, ruled out deficiency as the cause. Lastly, bone biopsy revealed no abnormalities, leading us to believe that the etiology was idiopathic and led to a diagnosis of ITP.^[16,23]^ This early diagnosis allows for prompt initiation of treatment for ITP, potentially leading to faster improvement in platelet count and minimizing the risk of complications associated with low platelet count, such as bleeding.

### 3.4. Differential diagnosis

Differentiating ITP from other causes of thrombocytopenia is critical to ensure appropriate management. ITP can be diagnosed with confidence by systematically ruling out secondary causes and microangiopathies. The lack of systemic symptoms, normal blood smear, and isolated thrombocytopenia in the absence of splenomegaly often supports the diagnosis. Differential diagnosis of ITP requires careful exclusion of other potential causes of thrombocytopenia, particularly in patients presenting with overlapping clinical manifestations. ITP is a diagnosis of exclusion because no definitive test can confirm it. Establishing ITP as a final diagnosis involves differentiating it from a variety of other hematologic, infectious, autoimmune, and drug-induced conditions.^[17]^

As previously mentioned, ITP is diagnosed by a low platelet count, with normal white and red blood cell counts. The absence of splenomegaly or systemic symptoms with normal bone marrow morphology in cases where a biopsy is performed, with the absence of definitive diagnostic markers, ruling out secondary causes, is essential. Drug-induced thrombocytopenia (DITP) may result from medications, such as heparin, quinine, and certain antibiotics, which can cause immune-mediated thrombocytopenia. In DITP, thrombocytopenia resolves upon drug discontinuation. The diagnosis of DITP can be confirmed using drug-specific antibodies (e.g., for heparin-induced thrombocytopenia). Another common cause of thrombocytopenia is infection with HIV, hepatitis C, and *H pylori*. Infectious thrombocytopenia is often accompanied by systemic signs of infection (e.g., fever and hepatosplenomegaly) and viral serology, and *H pylori* stool or urea breath tests are commonly used diagnostic tests. Autoimmune disorders, such as systemic lupus erythematosus and antiphospholipid syndrome, can also cause immune-mediated thrombocytopenia. These conditions are characterized by the presence of systemic autoimmune features (e.g., arthritis, rash, and recurrent thrombosis), which can be confirmed by positive antinuclear antibody, dsDNA antibodies, or antiphospholipid antibody tests. Lymphoproliferative disorders, such as chronic lymphocytic leukemia or lymphoma, may present with thrombocytopenia due to bone marrow infiltration or immune dysregulation, characterized by lymphadenopathy or abnormal lymphocyte count. Flow cytometry or bone marrow biopsy can provide a definitive diagnosis. Microangiopathies such as thrombotic thrombocytopenic purpura, caused by ADAMTS13 deficiency, leading to microangiopathic hemolytic anemia and thrombocytopenia with neurological symptoms, renal impairment, and schistocytes on a blood smear, can be ruled out using the ADAMTS13 activity assay. Hemolytic uremic syndrome is often triggered by Shiga toxin-producing *Escherichia coli* infection with hemolysis, thrombocytopenia, and acute kidney injury, which can be ruled out by stool culture for Shiga toxin-producing Escherichia coli. Bone marrow disorders, such as aplastic anemia, can present with thrombocytopenia associated with pancytopenia and can be diagnosed by hypocellular bone marrow biopsy. Myelodysplastic syndromes include clonal hematopoietic disorders that mainly affect elderly patients, and dysplastic blood cells and thrombocytopenia are ruled out by cytogenetic analysis of the bone marrow. Another cause of thrombocytopenia can be splenic sequestration, seen in conditions of splenomegaly associated with cirrhosis or hematologic malignancies, which can be diagnosed by imaging studies, such as ultrasound and CT scans. Pregnancy-associated thrombocytopenia includes gestational and preeclampsia/eclampsia or hemolysis/elevated liver enzymes with low platelets syndrome, diagnosed by low platelet counts, usually resolving postpartum for the mild gestational type or evidence of hemolysis, abnormally elevated liver function enzymes, and hypertension in more severe cases.^[24–26]^

### 3.5. Management

ITP management plans aim to increase platelet counts and lower the risk of bleeding. Observing patients with mild thrombocytopenia and minor bleeding symptoms may be more appropriate than seeking emergency medical attention in the absence of bleeding manifestations. However, IVIG and corticosteroids started to offer a quick increase in platelet counts due to the persistence of severe thrombocytopenia and the emergence of recurrent epistaxis in this case. The patient responded well to the treatment, resulting in a significant increase in platelet count. As a precautionary measure, corticosteroids are recommended as a maintenance therapy.^[27]^

The concurrent use of high-dose corticosteroids in patients with an excellent IVIG response reflects an effort to combine the rapid action of IVIG with the sustained immunosuppressive effects of corticosteroids. IVIG exerts its effects by saturating Fc receptors on splenic macrophages, thereby inhibiting Fc-mediated phagocytosis of antibody-opsonized platelets.^[28]^ Corticosteroids downregulate pro-inflammatory cytokines (e.g., IL-1, IL-6, and TNF-α) and promote T regulatory cell activity, contributing to overall immune suppression.^[29]^ This combination approach is supported by the different molecular mechanisms underlying their effects, and the goal of optimizing short- and long-term platelet stability, as summarized in Table 2.

**Table 2:** Difference between IVIG and corticosteroids.

Feature	IVIG	Corticosteroids
Onset of action	Rapid (1–3 days)	Delayed (2–7 days)
Primary mechanism	Fc receptor blockade	Reduced autoantibody synthesis
Duration of effect	Short-term (2–4 weeks)	Longer-lasting
Target	Splenic macrophages and B cells	T cells, macrophages, and autoantibodies
Indications	Emergency situations (e.g., bleeding)	Initial management of newly diagnosed ITP

ITP = immune thrombocytopenic purpura, IVIG = intravenous immunoglobulin.

### 3.6. Treatment protocols

In addition to first-line IVIG plus corticosteroid treatment, alternative therapies may be considered in refractory cases or when there are contraindications to first-line therapy. Alternatives include rituximab, thrombopoietin receptor agonists, and splenectomy.

Table 3 summarizes the key differences among the alternative treatments.

**Table 3:** Difference between each treatment option.

Treatment	Efficacy	Time to response	Route of administration	Main risks
Rituximab	40–60%	Weeks	IV infusion	Immunosuppression, infections
TPO-Ras	>70%	Days to weeks	Oral (eltrombopag) or SC	Thrombosis, hepatotoxicity
Splenectomy	60–80%	Immediate to weeks	Surgical	Surgical risks, infections

IV = intravenous, SC = subcutaneous, TPO-Ras = thrombopoietin receptor agonists.

Rituximab is a monoclonal antibody targeting CD20 on B cells that is used to suppress antibodies that are responsible for platelet destruction in ITP.^[30]^ Thrombopoietin receptor agonists, including eltrombopag and romiplostim, stimulate megakaryocyte production in bone marrow to enhance platelet production.^[29]^ Splenectomy is the definitive treatment option for ITP, particularly in chronic or refractory cases.^[30]^

It is essential to tailor the course of treatment based on the patient’s characteristics, the degree of thrombocytopenia, and the presence of bleeding signs. Immunosuppressive medications, splenectomy, and more recent targeted therapies such as thrombopoietin receptor agonists are among the various treatments available for patients with refractory or chronic ITP.^[29]^ This case report highlights the difficulties in diagnosing ITP when dealing with unusual clinical presentations. This highlights the necessity for a thorough evaluation that includes complete medical history, physical examination, and laboratory tests to make an accurate diagnosis. Tailor-made care techniques based on the patient’s clinical presentation and disease history are crucial for achieving positive results and preventing life-threatening consequences associated with severe thrombocytopenia in ITP.

### 3.7. Limitations

While this case report is elaborate and provides comprehensive information, the proposed mechanisms for this patient remain unclear without biomarker profiling. Long-term follow-up data would have provided more insights into the progression of chronic ITP, replacement rates, and the durability of remission.

## 4. Conclusion

This case report identifies that early diagnosis is possible owing to in-depth knowledge regarding all typical and atypical manifestations of ITP. ITP is a diagnostic challenge even for the most experienced clinicians because of its idiopathic nature and mix of typical and atypical manifestations, and the diagnosis requires a high degree of suspicion. ITP can be diagnosed with a simple blood picture, but clinically, to present it as a definitive diagnosis requires an intensive range of tests to exclude any other possibilities. Corticosteroids and IVIG are the most commonly used treatments for ITP. However, the introduction of newer immunotherapeutic drugs has led to the development of multiple treatment strategies, and splenectomy is considered only in rare cases. A delay in the diagnosis of ITP can lead to life-threatening bleeding complications. This case report demonstrates that early diagnosis is possible with high clinical suspicion, specialized treatment strategies tailored to the severity of ITP, and close follow-up, resulting in significantly better outcomes for patients.

## Author contributions

**Conceptualization:** Tirath Patel.

**Data curation:** Monica Kharat, Hitanshi Panchal, Nana Sardarova.

**Formal analysis:** Monica Kharat, Jabez David John, Nana Sardarova.

**Investigation:** Jabez David John, Gayatri Misra.

**Project administration:** Tirath Patel.

**Supervision:** Richard M. Millis.

**Validation:** Hitanshi Panchal, Muhammad Ahmed.

**Writing – original draft:** Tirath Patel, Jabez David John.

**Writing – review & editing:** Tirath Patel, Monica Kharat, Hitanshi Panchal, Gayatri Misra, Muhammad Ahmed, Richard M. Millis.
